# Caspase-7 Activation by the Nlrc4/Ipaf Inflammasome Restricts *Legionella pneumophila* Infection

**DOI:** 10.1371/journal.ppat.1000361

**Published:** 2009-04-03

**Authors:** Anwari Akhter, Mikhail A. Gavrilin, Laura Frantz, Songcerae Washington, Cameron Ditty, Dominique Limoli, Colby Day, Anasuya Sarkar, Christie Newland, Jonathan Butchar, Clay B. Marsh, Mark D. Wewers, Susheela Tridandapani, Thirumala-Devi Kanneganti, Amal O. Amer

**Affiliations:** 1 Division of Pulmonary, Allergy, Critical Care, and Sleep Medicine, Center for Microbial Interface Biology and the Department of Internal Medicine, Ohio State University, Columbus, Ohio, United States of America; 2 Department of Immunology, St Jude Children's Research Hospital, Memphis, Tennessee, United States of America; University of Toronto, Canada

## Abstract

*Legionella pneumophila* (*L. pneumophila*), the causative agent of a severe form of pneumonia called Legionnaires' disease, replicates in human monocytes and macrophages. Most inbred mouse strains are restrictive to *L. pneumophila* infection except for the A/J, Nlrc4^−/−^ (Ipaf^−/−^), and caspase-1^−/−^ derived macrophages. Particularly, caspase-1 activation is detected during *L. pneumophila* infection of murine macrophages while absent in human cells. Recent *in vitro* experiments demonstrate that caspase-7 is cleaved by caspase-1. However, the biological role for caspase-7 activation downstream of caspase-1 is not known. Furthermore, whether this reaction is pertinent to the apoptosis or to the inflammation pathway or whether it mediates a yet unidentified effect is unclear. Using the intracellular pathogen *L. pneumophila*, we show that, upon infection of murine macrophages, caspase-7 was activated downstream of the Nlrc4 inflammasome and required caspase-1 activation. Such activation of caspase-7 was mediated by flagellin and required a functional Naip5. Remarkably, mice lacking caspase-7 and its macrophages allowed substantial *L. pneumophila* replication. Permissiveness of caspase-7^−/−^ macrophages to the intracellular pathogen was due to defective delivery of the organism to the lysosome and to delayed cell death during early stages of infection. These results reveal a new mechanism for caspase-7 activation downstream of the Nlrc4 inflammasome and present a novel biological role for caspase-7 in host defense against an intracellular bacterium.

## Author Summary

*Legionella pneumophila* causes a severe form of pneumonia called Legionnaires' disease. In human macrophages, *L. pneumophila* establishes special vacuoles that do not fuse with the lysosome and grows intracellularly. However, in mouse macrophages, the bacteria are efficiently delivered to the lysosome for degradation. Importantly, caspase-1 is activated when *L. pneumophila* infects mouse macrophages, but not when it infects human cells. Caspase-1 activation promotes the fusion of the *L. pneumophila* vacuole with the lysosome and macrophage death. However, the caspase-1 substrate mediating such effects is unknown. Experiments performed *in vitro* demonstrate that caspase-7 is a substrate of caspase-1. Yet, it is not known if the reaction takes place within the macrophage, and it is unclear if it has any biological effect. In this study we show that, in mouse macrophages, caspase-7 is activated by *L. pneumophila* downstream of caspase-1 and requires the host receptors Nlrc4 and Naip5. Remarkably, caspase-7 activation during *L. pneumophila* infection restricts growth by promoting early macrophage death and efficient delivery of the organism to the lysosome. Consequently, *L. pneumophila* grows in the macrophages and the lungs of caspase-7^−/−^ mice. Therefore, we demonstrate a novel caspase-7 activation pathway that contributes to the restriction of *L. pneumophila* infection.

## Introduction

Caspases are a family of cysteine proteases expressed as inactive pro-enzymes that play a central role in most cell death pathways leading to apoptosis. However, growing evidence implicates caspases in non-apoptotic functions [1]–[4]. Eleven genes were found in the human genome to encode 11 human caspases, whereas 10 genes were found in the mouse genome to encode 10 murine caspases. The human caspase-4 and -5 are functional orthologs of mouse caspase-11 and -12. The remaining caspases which share same nomenclature in human and mouse are functional orthologs of each other [1]. On the basis of their biological functions, caspases can be classified into three groups: inflammatory caspases like caspase-1, -4, -5, -11 and -12, initiator caspases like caspase-2, -8, -9, and -10, and effector caspases like caspase-3, -6, -7 and -14 [2],[4]. Caspase-1 activation mediates the maturation of the proinflammatory cytokines interleukin-1 beta (IL-1β), IL-18 and possibly IL-33 [5],[6]. Activation of caspase-1 is mediated within the inflammasome complex that is assembled when pathogen-associated molecular patterns (PAMPs) are sensed in the cytosol by special host receptors. These cytosolic receptors belong to the nucleotide binding oligomerization domain-leucine rich repeat proteins (NOD-like-receptors or CATERPILLAR family of proteins) [7]–[12]. A variety of pathogens such as *Shigella*, *Francisella*, *Salmonella*, *Listeria*, *Pseudomonas*, *Escherichia coli* and *Legionella* activate caspase-1, engaging different NOD-like-receptors [13]–[18].

*L. pneumophila* is an intracellular bacterium and the causative agent of Legionnaires' pneumonia [19]. The ability of *L. pneumophila* to cause pneumonia is dependent on its tendency to invade and multiply within human macrophages [20]–[23]. Once phagocytized, the bacteria reside in specialized vacuoles [20]–[26]. The *L. pneumophila-*containing phagosome does not fuse with the lysosome and instead acquires vesicles from the endoplasmic reticulum (ER) [20]–[26]. Within this vacuole, *L. pneumophila* multiply exponentially [27]. In contrast, macrophages from most mouse strains are restrictive to *L. pneumophila* infection. Within mice cells, *L. pneumophila* flagellin is sensed by Nlrc4 leading to the activation of caspase-1 [7], [28]–[30], whereas in human macrophages, caspase-1 is not activated in response to *L. pneumophila.* Caspase-1 activation in mouse macrophages is accompanied with *L. pneumophila* restriction due to the delivery of organisms to the lysosome for degradation and early macrophage death [28],[31]. Furthermore, pharmacological inhibition of caspase-1 in wild-type macrophages allows more *L. pneumophila* replication [28],[31],[32]. Accordingly, mouse macrophages that do not activate caspase-1 in response to *L. pneumophila* such as Nlrc4^−/−^ and caspase-1^−/−^ cells are permissive to infection [28],[31]. A/J mice and their derived macrophages are also permissive to *L. pneumophila* intracellular replication despite caspase-1 activation [28], [29], [33]–[36]. The downstream mechanism responsible for the permissiveness of macrophages lacking Nlrc4, caspase-1 or functional Naip5 is still unclear.

*In vitro* experiments revealed that caspase-1 directly processed procaspase-3 and -7 [37],[38]. Nevertheless, the biological role of this activation is unknown. Furthermore, whether this reaction is pertinent to the apoptosis or to the inflammation pathway or whether it mediates a yet unidentified effect is not clear.

Here we demonstrate that caspase-7, but not caspase-3, was activated in restrictive wild-type mouse macrophages by *L. pneumophila*. Caspase-7 activation by low multiplicity of *L. pneumophila* infection was dependent on Nlrc4, caspase-1 and functional Naip5. Such activation was accompanied by enhanced fusion of the *L. pneumophila*-containing phagosome with the lysosome and early death of infected cells resulting in restriction of infection in wild-type macrophages. The activation of caspases-8 and -9 which are involved in caspase-7 activation in response to apoptotic signals was not necessary for *L. pneumophila*-mediated caspase-7 activation. In contrast to caspase-7, caspase-3 was not activated by *L. pneumophila* in wild-type macrophages and its absence did not affect the activation of caspase-7 or the intracellular fate of the organism. The effect of caspase-7 activation on *L. pneumophila* growth was independently of IL-1β and IL-18. Remarkably, caspase-7 activation also controlled the growth of the pathogen within the murine lungs *in vivo*.

Therefore, our data identify a previously uncharacterized signaling pathway for caspase-7 activation through Nlrc4, caspase-1 and Naip5. We also demonstrate a new role for caspase-7 in host defense against an intracellular bacterium. These findings may be valuable in the design of compounds that could restrict the growth of not only *L. pneumophila* but also other organisms that tend to avoid lysosomal fusion.

## Results

### Caspase-7 is activated by wild-type *L. pneumophila*

**Figure 1:**
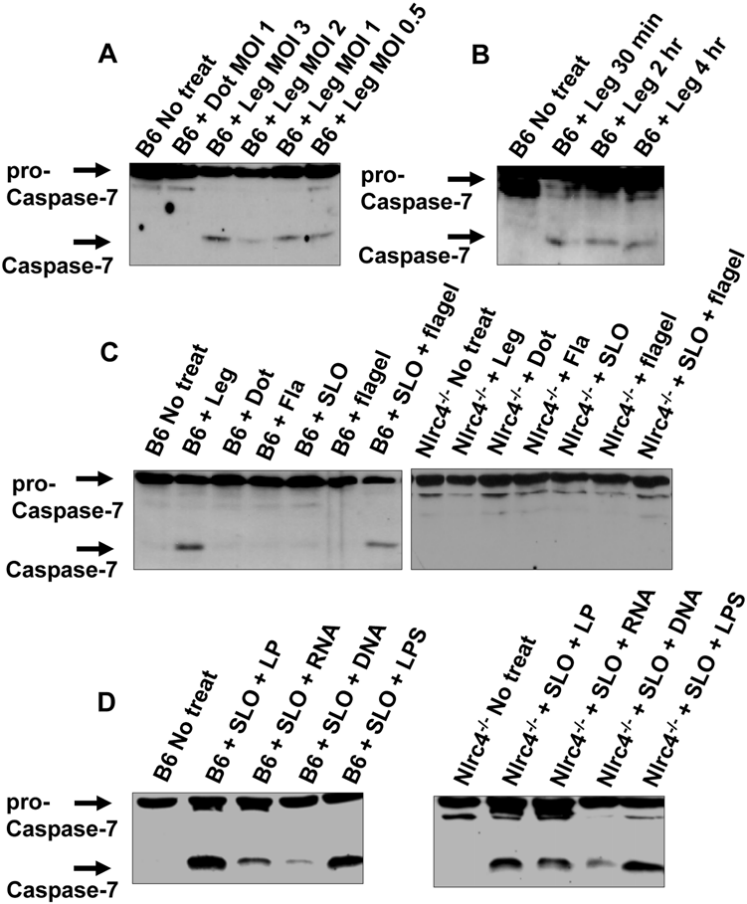
The host protein Nlrc4 and bacterial flagellin are required for the induction of caspase-7 activation by *L. pneumophila.* (A) Wild-type C57BL/6 (B6) derived macrophages were not treated (No treat) or infected with wild-type *L. pneumophila* (Leg) at different multiplicities of infection (MOI) (3-0.5) or with *L. pneumophila* type IV secretion mutant (Dot) at an MOI of 1 for 2 hrs. (B) B6 derived macrophages were infected with Leg at an MOI of 0.5 for different durations. (C,D) B6 and Nlrc4^−/−^ macrophages were infected with Leg, the Dot mutant, the mutant lacking flagellin (Fla) (MOI of 0.5) or treated with streptolysin O (SLO) alone, flagelin alone (flagel), or SLO and purified flagel (C), or treated with SLO and bacterial lipoprotein (LP), bacterial RNA, bacterial DNA, or bacterial lipopolysaccharide (LPS) (D). (A–D) Immunoblots were developed with anti-caspase-7 antibody and are representative of more than three independent experiments.

*L. pneumophila* infection leads to caspase-1 activation in macrophages. The activation of caspase-1 is accompanied by restriction of *L. pneumophila* growth in macrophages and in mice [28],[31]. However, the downstream signaling pathway involved in the control of *L. pneumophila* growth is not known. *In vitro* studies suggested that caspase-1 can cleave caspase-7 and caspase-3 [37],[38]. However, it is not known if this reaction takes place *in vivo* during *L. pneumophila* infection. Therefore, we investigated whether caspase-7 and caspase-3 are cleaved within wild-type C57BL/6 macrophages upon *L. pneumophila* infection. Assessment of different multiplicity if infection (MOI) revealed that caspase-7 was activated at MOI ranging from 0.5 to 20 within 2 hours of infection. Caspase-1 activation was determined by the detection of the corresponding mature subunits in cell extracts in western blots using specific anti-caspase-1 antibodies (Figure 1A and 1B and Figure S1A). Infection of wild-type murine macrophages was accompanied by caspase-7 activation only in the presence of a functional Dot/Icm type IV secretion system, a bacterial apparatus that injects bacterial products into the host cytosol (Figure 1A and 1C). The bacteria induced proteolytic cleavage of pro-caspase-7 within 30 minutes even at low MOI (Figure 1B). Therefore, *L. pneumophila* activates caspase-7 in macrophages in a Dot/Icm-dependent manner.

### Bacterial flagellin mediates caspase-7 activation via the host protein Nlrc4

Since *L. pneumophila* flagellin triggers caspase-1 activation via the NOD-like receptor Nlrc4 [7],[28], we tested whether a *L. pneumophila* mutant lacking flagellin (Fla) induced caspase-7 activation. We infected wild-type macrophages with *L. pneumophila* or with the Fla mutant lacking flagellin and examined the activation of caspase-7 (Figure 1C). To ensure equal internalization of wild-type and flagellin-deficient *L. pneumophila*, infections were followed by mild centrifugation to enhance contact between macrophages and mutant bacteria. Under these conditions, we recovered equal numbers of bacteria at 1 hour post-infection (data not shown). Unlike wild-type bacteria, the *L. pneumophila* Fla mutant failed to induce caspase-7 activation even at high MOI (Figure 1C, Figure S1B, and Figure S1C). Since flagellin is sensed by the host receptor Nlrc4, we tested if caspase-7 activation by *L. pneumophila* requires Nlrc4. Caspase-7 activation by wild-type *L. pneumophila* was abrogated in macrophages lacking Nlrc4 (Figure 1C). To verify the role of flagellin in caspase-7 activation, purified bacterial molecules were delivered intracellularly using streptolysin O (SLO), a protein that allows delivery of exogenous molecules into the cytosol of living cells [28],[39]. Bacterial flagellin, bacterial lipoproteins (LP), RNA, DNA, or lipopolysaccharide (LPS) activated caspase-7 when delivered to the cytosol using SLO (Figure 1C and 1D). However, flagellin required Nlrc4 for caspase-7 activation (Figure 1C and 1D). The low concentration and short time of SLO treatment used to deliver flagellin to the cytosol did not activate caspase-7 in the absence of bacterial ligands (Figure 1C). Therefore, caspase-7 activation by *L. pneumophila* is mediated by the bacterial flagellin through the host sensor Nlrc4.

To rule out the contribution of Toll-Like Receptor (TLR) signaling in caspase-7 activation by *L. pneumophila*, macrophages lacking the TLR adaptor molecules MyD88 or TRIF were infected and examined for activation of caspase-7. The lack of MyD88 or TRIF did not prevent caspase-7 activation by *L. pneumophila* (Figure S2A and Figure S2B). These results indicate that the sensing of flagellin through Nlrc4 mediates caspase-7 activation by *L. pneumophila* independently of TLRs.

### Caspase-7 activation by *L. pneumophila* infection is dependent on caspase-1, but not caspase-3

**Figure 2:**
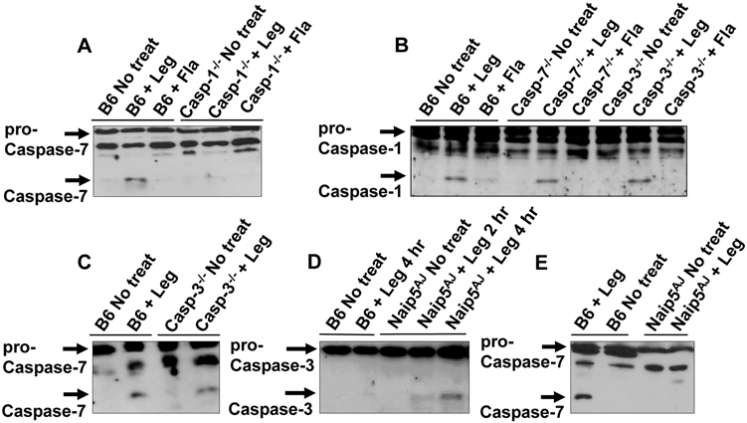
Caspase-1 and Naip5 are required for caspase-7 activation by *L. pneumophila.* (A) Wild-type C57BL/6 (B6) and caspase-1^−/−^ (casp-1^−/−^) derived macrophages were not treated (No treat) or infected with *L. pneumophila* (Leg), or the mutant lacking flagellin (Fla) for 2 hr. Cell lysates were analyzed by western blot with anti–caspase-7 antibodies. (B) B6, caspase-7^−/−^ (casp-7^−/−^), and caspase-3^−/−^ (casp-3^−/−^) macrophages were treated with wild-type Leg or with Fla mutant, then cell lysates were analyzed by western blot with anti–caspase-1 antibodies. (C) B6 and casp-3^−/−^ were infected with Leg, then cell lysates were examined by western blots with anti–caspase-7 antibodies. (D,E) B6– and A/J–derived macrophages (Naip5^AJ^) were infected or not with Leg for times indicated, then cell lysates were examined by western blot with anti–caspase-3 (D) or –caspase-7 (E) antibodies.

Nlrc4 is indispensable for caspase-1 and caspase-7 activation by *L. pneumophila.* However, it is not clear if caspase-1 activation is upstream of caspase-7 as suggested by the *in vitro* experiments [38]. To assess the role of caspase-1 in caspase-7 activation during early stages of infection, caspase-1^−/−^ macrophages were infected with low MOI of *L. pneumophila*. Particularly, caspase-7 activation by *L. pneumophila* was abolished in the absence of caspase-1 (Figure 2A), whereas caspase-1 activation by *L. pneumophila* did not require caspase-7 (Figure 2B). This result suggests that caspase-7 is downstream of the caspase-1 activation pathway. However, at high MOI caspase-7 was activated independently of caspase-1 (Figure S1B).

To verify if the enzymatic activity of caspase-1 contributes to the induction of caspase-7 activation by low MOI of *L. pneumophila*, we inhibited caspase-1 activity with the caspase-1 inhibitor Z-YVAD-FMK and examined caspase-7 activation upon *L. pneumophila* infection. Pharmacological inhibition of caspase-1 abolished caspase-7 activation by *L. pneumophila* (Figure S3A). Taken together, our data show that caspase-1 enzymatic activity is necessary for caspase-7 activation by *L. pneumophila* in macrophages.

**Figure 3:**
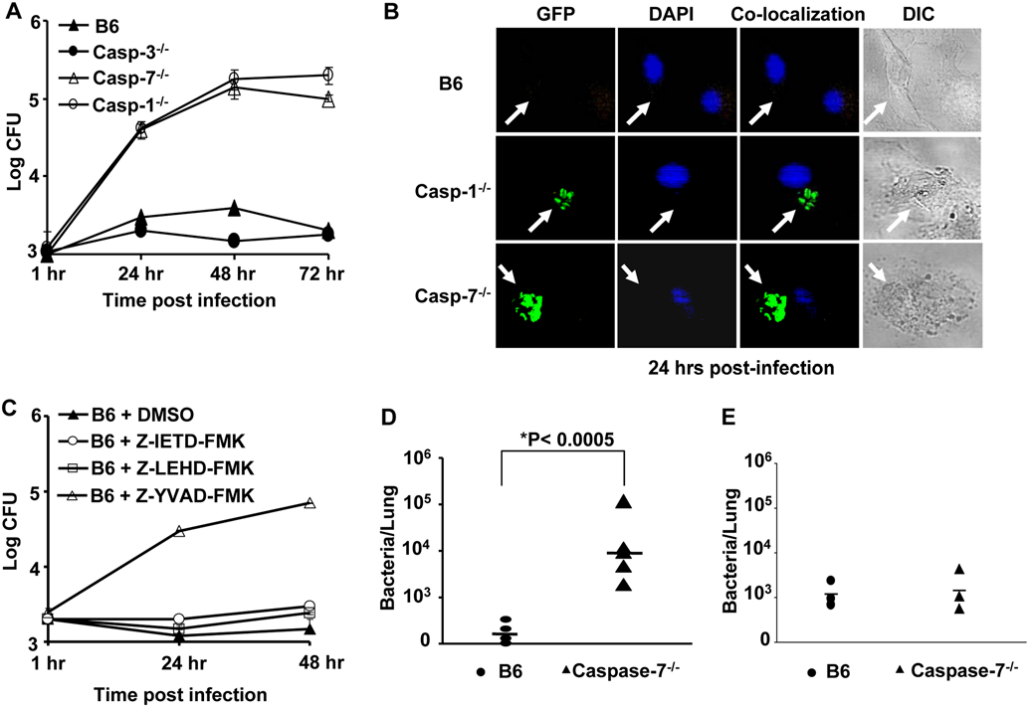
Caspase-7 restricts *L. pneumophila* replication in macrophages and in mice. (A) Wild-type C57BL/6 (B6), caspase-3^−/−^ (casp-3^−/−^), caspase-7^−/−^ (casp-7^−/−^), or caspase-1^−/−^ (casp-1^−/−^) macrophages were infected with wild-type *L. pneumophila*, then colony forming units (CFU) were scored at indicated time points. The results represent the mean of four independent experiments ±SD. (B) B6, casp-1^−/−^, or casp-7^−/−^ macrophages were infected with GFP–expressing *L. pneumophila* for 24 hrs and examined by fluorescence microscopy. (C) B6 macrophages were treated or not with 50 µM caspase-1 inhibitor (YVAD), caspase-8 inhibitor (IETD), caspase-9 inhibitor (LEHD), or caspase-3 inhibitor or dimethylsulfoxide alone (DMSO) 45 min before infection with *L. pneumophila*. Cells were lysed and the number of colony forming units (CFU) was quantified at 1, 24, and 48 hrs post infection. The results represent the mean of three independent experiments ±SD. (D) B6 and caspase-7^−/−^ mice received 10^6^ wild-type *L. pneumophila* intra-tracheally. Lungs were homogenized and plated for CFUs at 96 hrs post-infection. Data were analyzed by Student's t-test. *, *P* value≤0.05. (E) B6 and caspase-7^−/−^ mice were infected with *L. pneumophila* for 6 hrs and lungs were examined for bacterial load as described in D.

Caspase-7 and caspase-3 are activated during apoptosis via the intrinsic and extrinsic pathways through caspase-8 and -9 [3]. First, to understand the role of caspase-3 in *L. pneumophila*-mediated caspase-7 activation, caspase-3^−/−^ macrophages were infected with *L. pneumophila* and examined for the cleavage of caspase-1 and caspase-7. Our data demonstrate that the absence of caspase-3 had no effect on pathogen-mediated activation of caspase-1 (Figure 2B) or of caspase-7 (Figure 2C). These data support the fact that caspase-3 was not activated by *L. pneumophila* in wild-type C57BL/6 macrophages although activated in A/J-derived macrophages (Figure 2D). Second, to test the role of the initiator caspase-8 and -9 in caspase-7 activation by *L. pneumophila* infection, the pathogen was added to macrophages pretreated with the caspase-8 inhibitor (Z-IETD-FMK), or caspase-9 inhibitor (Z-LEHD-FMK), then cell lysates were examined for caspase-7 activation. In contrast to caspase-1 inhibition, the pharmacological inhibition of caspase-8 or -9 did not compromise caspase-7 activation by *L. pneumophila* (Figure 3A). However, inhibition of caspase-8 and -9 activities compromised caspase-7 activation induced by the apoptosis inducer doxorubicin (Figure S3B). These results indicate that caspase-7 activation by *L. pneumophila* is independent of caspase-8 or -9.

### Naip5 is essential for caspase-1–mediated activation of caspase-7 by *L. pneumophila*

With the exception of the A/J mouse, most mouse strains are resistant to *L. pneumophila* [20],[36],[40]. The permissiveness of the A/J mouse is attributed to a polymorphism in the gene encoding the neuronal apoptosis inhibitory protein (*Naip5*) [34],[35],[41]. The susceptibility of A/J-derived macrophages to *L. pneumophila* is independent of caspase-1 activation since the levels of activation in the presence of wild-type and A/J-derived Naip5 are comparable [28],[29],[33]. Given that caspase-7 activation is mediated downstream of caspase-1 in wild-type macrophages, we tested if Naip5 plays a role in caspase-7 activation by *L. pneumophila*. Caspase-7 activation by *L. pneumophila* was compromised in macrophages derived from A/J-derived macrophages (Naip5^AJ^) (Figure 2E). Similar results were obtained using transgenic C57BL/6 mice harboring the mutant A/J-derived Naip5 (data not shown) [42]. These findings suggest that *L. pneumophila*–mediated caspase-7 activation downstream of the caspase-1 inflammasome requires Naip5.

### Caspase-7 activation restricts *L. pneumophila* replication in macrophages and in mice

Caspase-1 activation restricts *L. pneumophila* intracellular survival and replication [28],[31]. However, the downstream effectors responsible for the control of *L. pneumophila* growth are still unknown. Given that caspase-7 activation by *L. pneumophila* is dependent on caspase-1 activation, we investigated if caspase-7 controls *L. pneumophila* replication. Wild-type, caspase-3^−/−^, caspase-7^−/−^ and caspase-1^−/−^ macrophages were infected with *L. pneumophila* and intracellular bacterial replication was evaluated by quantifying the colony forming units (CFUs) throughout 72 hours of infection and by microscopy after 24 hours. Remarkably, macrophages lacking caspase-7 allowed substantial *L. pneumophila* replication when compared to wild-type cells (Figure 3A and 3B). The number of CFUs recovered from caspase-7^−/−^ macrophages were comparable to the number recovered from their caspase-1^−/−^ counterparts (Figure 3A and 3B). Peritoneal macrophages from caspase-7^−/−^ mice were also highly permissive for *L. pneumophila* replication (data not shown).

To confirm the role of caspase-7 in *L. pneumophila* restriction, caspase-7^−/−^ macrophages were complemented with caspase-7 plasmid and examined for the correlation between caspase-7 expression and bacterial replication. The delivery of a caspase-7 plasmid to primary caspase-7^−/−^ macrophages was performed using transfection (Superfect) or nucleofection (Amaxa). Although caspase-7 was expressed using both techniques, nucleofection was detrimental to the cells (Figure S4A and data not shown). Expression of caspase-7 using Superfect was sufficient to restore the ability of murine macrophages to restrict *L. pneumophila* growth (Figure S4A and Figure S4B). The expression of the control plasmid carrying the red fluorescent protein (RFP) or green florescent protein (GFP) by the same techniques did not alter the permissiveness of the caspase-7^−/−^ macrophages to *L. pneumophila* (Figure S4B and data not shown).

Since caspase-1 activity was necessary for *L. pneumophila*-mediated caspase-7 activation (Figure S3A), we tested if inhibition of caspase-1 activity permitted more bacterial replication. Distinctly, the inhibition of caspase-1 activity by Z-YVAD-FMK allowed more bacterial growth in wild-type B6 macrophages (Figure 3C). Unlike caspase-1 inhibition, the inhibition of caspase-8, -9 or -3 did not allow *L. pneumophila* replication in B6 macrophages (Figure 3C). Therefore, caspase-1 enzymatic activity is required for *L. pneumophila* growth restriction in macrophages. Unlike caspase-7, and despite the suggested similarity in small substrate specificity, the absence of caspase-3 had no effect on *L. pneumophila* growth in macrophages (Figure 3A).

Since Legionnaires' disease is caused by the replication of *L. pneumophila* in alveolar macrophages, we investigated if caspase-7 regulates bacterial growth within the lungs of mice *in vivo*. Wild-type and caspase-7^−/−^ mice were infected intratracheally with 1×10^6^ CFU of *L. pneumophila* and the number of bacteria in the lungs was determined at 96 hours post-infection (Figure 3D). The lungs of caspase-7^−/−^ mice supported substantially more *L. pneumophila* CFUs than their wild-type counterparts (Figure 3D), although initial bacterial counts in the lungs were identical (Figure 3E). Therefore, these results indicate that caspase-7 restricts *L. pneumophila* replication both *in vitro* and *in vivo.*

### Caspase-1–dependent caspase-7 activation contributes to *L. pneumophila* phagosome maturation in macrophages

**Figure 4:**
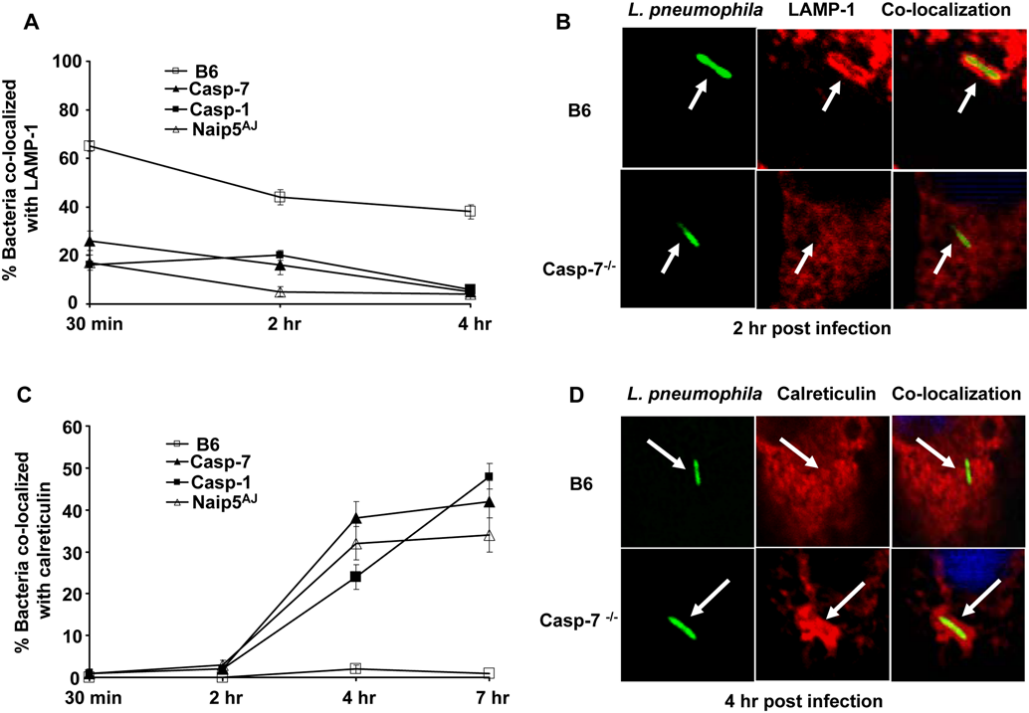
*L. pneumophila*–containing phagosomes avoid the fusion with the lysosome in caspase-7^−/−^, caspase-1^−/−^, and A/J–derived macrophages but not in wild-type macrophages. Macrophages from wild-type C57BL/6 (B6), caspase-7^−/−^ (casp-7^−/−^), caspase-1^−/−^ (casp-1^−/−^), or A/J mice were seeded on cover slips and infected with *L. pneumophilla.* The internalized bacteria were quantified for co-localization with the late endosomal marker LAMP-1 (A and B), or for the localization with the endoplasmic reticulum marker calreticulin at times indicated (C and D). Data represent the mean of three independent experiments ±SD.

In wild-type macrophages, a fraction of phagocytized *L. pneumophila* are contained inside phagosomes that rapidly fuse with lysosomes [22],[43],[44]. To determine the mechanism by which caspase-7 controls *L. pneumophila* growth in macrophages, we observed the trafficking of the organism intracellularly. First, we examined the rate of acquisition of the lysosomal-associated membrane protein-1 (LAMP-1) by phagosomes harboring *L. pneumophila*. Whereas, in wild-type macrophages, greater than 60% of the phagosomes containing the pathogen co-localized with LAMP-1 within 30 min post-infection, less than 30% of the *L. pneumophila* containing vacuoles in caspase-7^−/−^, caspase-1^−/−^ and A/J-derived macrophages acquired LAMP-1 (Figure 4A and 4B).

Next, to test if caspase-7^−/−^ macrophages contained a general defective phagosome-lysosome fusion function, we examined the ability of the non-pathogenic type IV secretion mutant (Dot) to traffic to the lysosome. In contrast to pathogenic wild-type *L. pneumophila*, caspase-7^−/−^ macrophages efficiently delivered the Dot mutants to LAMP-1 labelled compartments within 2 hours of internalization (Figure S5A). Then, in order to assess the role of caspase-7 in mediating *L. pneumophila* degradation [28],[45], macrophages were infected with *L. pneumophila*, and the intact rod-shaped and degraded distorted-shaped bacteria were differentiated by labelling with anti-*L. pneumophila* antibody as previously described (Figure S5B). In wild-type macrophages, by 2 hours post-infection, about 40% of internalized bacteria lost their rod-shaped contour and were degraded into multiple small rounded particles compared to only 10 % in caspase-7^−/−^ macrophages (Figure S5B).

Given that the *L. pneumophila*-containing phagosome in permissive cells acquires ER-derived vesicles [20],[22],[26], we examined the recruitment of calreticulin, an ER protein, to phagosomes harboring *L. pneumophila*. By 4 hours post-infection, around 35% of the bacteria co-localized with calreticulin in caspase-7^−/−^ macrophages, whereas less than 5% bacteria associated with the ER marker in B6 macrophages (Figure 4C and 4D). The trafficking of *L. pneumophila* in caspase-7^−/−^ macrophages was comparable to that in the caspase-1^−/−^ and A/J-derived counterparts (Figure 4A and 4C). Therefore, caspase-7 activation promotes the fusion of the *L. pneumophila*-containing phagosome with the lysosome mediating the degradation of the pathogen.

### Caspase-7 activation and restriction of *L. pneumophila* growth is independent of IL-1β and IL-18

Caspase-7 activation by *L. pneumophila* is regulated by caspase-1 which also regulates the maturation of the pro-inflammatory cytokines IL-1β and IL-18 [2],[5]. Our data show that caspase-7 activation is mediated by Nlrc4 and caspase-1 (Figure 1C and Figure 2A and 2E). To examine the possibility that caspase-7 is downstream of IL-1β and IL-18, we infected wild-type and IL-1β/IL-18 double knockout-derived macrophages with *L. pneumophila* and examined the activation of caspase-7. The absence of IL-1β and IL-18 did not prevent caspase-7 activation by *L. pneumophila* infection (Figure S6A). Next, to test if IL-1β and IL-18 control *L. pneumophila* growth, macrophages lacking both cytokines were infected and bacterial growth was quantified throughout 72 hours. Cells lacking IL-1β and IL-18 did not allow *L. pneumophila* replication intracellularly when compared with their caspase-7^−/−^ and A/J-derived counterparts (Figure S6B). To further understand the role of IL-1β and IL-18 in infection, recombinant mouse IL-1β (rIL-1β) or IL-18 (rIL-18) was added to B6 and caspase-7^−/−^ macrophages upon *L. pneumophila* infection. Exogenous cytokines had no effect on the growth of the pathogen whether caspase-7 was present or not (Figure S6C). Next, to examine the role of IL-1β and IL-18 receptors during *L. pneumophila* infection, corresponding antibodies to IL-1 receptor and IL-18 receptor were added during infection. The intracellular growth of *L. pneumophila* in macrophages in the presence of the antibodies was identical to that of untreated cells (Figure S6C). Our data demonstrate that despite being downstream of caspase-1, IL-1β and IL-18 do not play a role in caspase-7 activation by *L. pneumophila* or restriction of macrophage infection.

To determine if caspase-7 is upstream of IL-1β and IL-18 and hence controls their activation, we examined IL-1β and IL-18 release in culture supernatants of wild-type and caspase-7^−/−^ macrophages in response to different organisms. IL-1β was released by *L. pneumophila* in the presence or absence of caspase-7 after 24 hours of infection. However, IL-18 was barely detected in both macrophage cell types in response to *L. pneumophila* (data not shown). Similarly, within 24 hrs of infection, IL-1β was released by wild-type and caspase-7^−/−^ macrophages in response to *Salmonella typhimurium* (*Salmonella*) (Figure S7A and Figure S7B). These results demonstrate that caspase-7 does not regulate the caspase-1–dependent inflammatory substrates IL-1β or IL-18.

### Caspase-7^−/−^ macrophages delay apoptosis during early stages of infection by *L. pneumophila*

Our data show that at physiological stages of infection, *L. pneumophila* leads to caspase-7 activation in a caspase-1–dependent manner (Figure S1). Nevertheless, when high numbers of organisms invade the macrophage, caspase-7 is induced independent of caspase-1.

**Figure 5:**
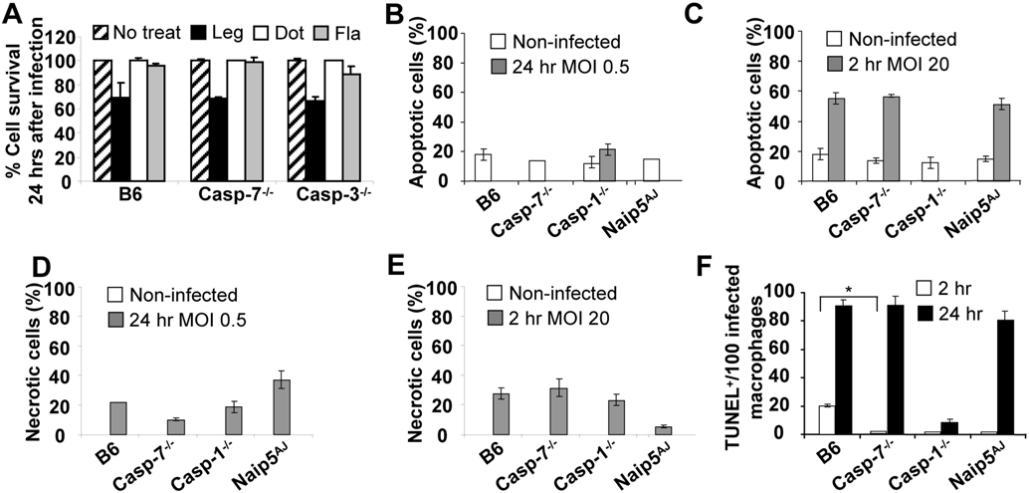
The role of caspase-1 and -7 in *L. pneumophila*–induced apoptosis at different multiplicities of infection (MOI). (A) Wild-type C57BL/6 (B6), casp-7^−/−^, and casp-3^−/−^ macrophages were not treated (striped bars), or treated with wild-type *L. pneumophila* (black bars), type IV secretion mutant (white bars), or flagellin mutant (grey bars) at MOI of 0.5 for 24 hrs, then percent cell survival was measured by LDH release from overall population of macrophages. Analysis of apoptosis (B and C) or necrosis (D and E) of macrophages not infected (white bars) or infected (grey bars) with wild-type *L. pneumophila* at an MOI of 0.5 for 24 hrs (B and D) or MOI of 20 for 2 hrs (C and E) by ELISA photometric enzyme immunoassay analysis of the cytoplasmic (apoptosis) and extracellular (necrosis) histone-associated-DNA-fragments. (F) Microscopic single cell analysis of apoptosis by TUNEL staining after 2 hrs (white bars) and 24 hrs (black bars) of infection with wild-type *L. pneumophila* at MOI of 0.5. The y axis represents the number of TUNEL positive (TUNEL^+^) macrophages among 100 infected macrophages. *, *P* value≤0.05. (A–F) The results represent the mean of three independent experiments ±SD.

It has been shown that macrophage cell death contributes to the restriction of *L. pneumophila* infection [44],[46]. Therefore, we examined the role of caspase-7 in induction of cell death during *L. pneumophila* infection at different MOIs and durations of infection. First we measured LDH release in the overall population of macrophages infected with low MOI for 24 hrs. Minimal cell death was detected in response to wild-type organism in infected macrophages whether they expressed caspase-7 or not (Figure 5A).

To better understand the role of caspase-7 in *L. pneumophila*-mediated cell death, we measured macrophage apoptosis and necrosis in the overall population of macrophages by determining the cytoplasmic (apoptosis) and extracellular (necrosis) histone-associated-DNA-fragments during low and high MOI. Low MOI of *L. pneumophila* infection lead to minimal apoptosis after 24 hrs of infection in WT macrophages and in those lacking caspase-7, -1, -3, or functional Naip5 (Figure 5B). However, at high MOI, *L. pneumophila* induced significant apoptosis within 2 hrs of infection in all macrophages except those lacking caspase-1 (Figure 5C). Indeed, after 24 hrs of high MOI, all macrophages were apoptotic irrespective of their type (data not shown).

At low MOI, around 20% of macrophages were necrotic after 24 hrs of infection (Figure 5D). The high bacterial MOI did not dramatically increase the number of necrotic cells within 2 hrs of infection (Figure 5E).

To follow the role of apoptosis in *L. pneumophila* infection, apoptosis of individual cells after labeling of DNA strand breaks (TUNEL) was quantified by fluorescence microscopy. We scored 100 infected cells and quantified how many of those were TUNEL positive. Within 2 hrs of infection at low MOI, no more than 2% of infected macrophages lacking caspase-1, -7 or functional Naip5 were TUNEL positive whereas 20% infected wild-type macrophages were apoptotic (Figure 5F). After 24 hrs of infection, the majority of infected wild-type, caspase-7^−/−^ and A/J-derived macrophages were apoptotic while infected caspase-1^−/−^ macrophages did not show signs of apoptosis even when harboring several bacteria (Figure 5F). Remarkably, after the 24 hrs infection, many wild-type B6 macrophages that did not seem to harbor bacteria also underwent apoptosis (data not shown). Therefore, infected wild-type macrophages respond to *L. pneumophila* infection by early apoptosis which render them unsuitable for optimal bacterial replication.

Then, to investigate the role of apoptosis *in vivo*, we infected wild-type, caspase-1^−/−^, caspase-7^−/−^ and A/J-derived macrophages intratracheally for three days. Then, harvested infected lung sections were stained with TUNEL to detect apoptotic cells. The degree of apoptosis observed in lung tissues was comparable among different infected lungs (Figure S8). These results suggest that apoptosis may not play a major role in *L. pneumophila* infection *in vivo* especially at latter stages of infection.

### Caspase-1 and caspase-7 activation are not detected in human monocytes in response to *L. pneumophila* infection

Human monocytes are permissive to *L. pneumophila* [20],[47]. The failure of human cells to restrict *L. pneumophila* infection is still under extensive studies. Remarkably, Caspase-1 activation is not detected during *L. pneumophila* infection of human cells [48]. To investigate the role of caspase-7 in human cells, we examined caspase-1 and capsase-7 activation in fresh human monocytes infected with *L. pneumophila* or with *Salmonella*. Caspase-1 was strongly activated in response to *Salmonella* but not in response to *L. pneumophila*. Similarly, caspase-7 was activated during *Salmonella* infection but not during *L. pneumophila* infection (Figure S9A and Figure S9B). Therefore, caspase-1 and caspase-7 are not activated in human monocytes upon *L. pneumophila* infection.

Taken together, our data show that the lack of caspase-7 activation is associated with permissiveness to *L. pneumophila* infection in mice and in humans.

## Discussion

The ability of *L. pneumophila* to survive in human cells presents a challenge to host defense. One common strategy for the host to deal with intracellular infection is to eliminate infected cells by caspase-mediated apoptosis. However, emerging reports demonstrate new functionally distinct roles for executioner caspases independent of cell death [1],[49]. Another strategy for eliminating intracellular bacteria is to deliver them to the lysosome for degradation. However, several pathogens have developed ways to deter such fate [43], [50]–[52].

Here we reveal a novel role for caspase-7 in host defense against *L. pneumophila*. We have identified a new activation pathway for caspase-7 that requires Nlrc4 and caspase-1, independent of the classical apoptosis pathway employing caspase-8 and -9. We also show that Naip5 contributes to caspase-7 activation downstream of caspase-1 during physiological levels of infection.

Caspase-7 activation restricts *L. pneumophila* infection *in vitro* and *in vivo*. This restriction is lost in caspase-7^−/−^ macrophages, but restored after expression of caspase-7 via transfection (Figure S4). Taken together, the role of caspase-7 in restriction of *L. pneumophila* infection is confirmed, and the possibility that other molecules that may cause permissiveness to *L. pneumophila* such as Nramp-1 or Naip5 are responsible is ruled out [34],[40].

To recognize the role of caspase-7 during different stages of infection and different MOI, we pursued macrophage infections with low MOI (0.5) or high MOI (20). Under low MOI, caspase-7 activation by *L. pneumophila* required Nlrc4 and caspase-1 (Figures 1 and 2). It is possible that caspase-1 is required for the proper assembly of the Nlrc4-inflammasome while the cleavage of caspase-7 is mediated by another unidentified molecule. This possibility was deemed unlikely since the pharmacological inhibition of caspase-1 activity abolished caspase-7 activation in response to *L. pneumophila* (Figure S3) and because caspase-1 directly cleaves pro-caspase-7 *in vitro* [38].

In addition to flagellin, bacterial ligands not sensed by Nlrc4 but known to activate caspase-1 through other NOD-like receptors also led to caspase-7 activation, suggesting that this may apply to other inflammasome complexes (Figure 1D). The role of caspase-7 activation in response to organisms that engage different inflammasomes remains to be elucidated.

*L. pneumophila*-triggered caspase-7 activation is distinct from the activation seen during apoptosis, as inhibition of caspases-8 or -9 did not prevent activation following *L. pneumophila* infection (Figure S3), nor did it permit additional bacterial growth (Figure 3C). Our results show that, along with taking part in apoptosis, caspase-7 activation performs an additional unrecognized role.

The route of intracellular trafficking of *L. pneumophila* affects at least in part, the outcome of infection [20],[23],[25],[26],[47],[53],[54], but the factors that dictate this route are not very well understood. Here we show that in caspase-7^−/−^ macrophages, only 20% of the internalized *L. pneumophila* were delivered to the lysosome as early as 30 min after infection. Within 4 hrs, less than 10% of the bacteria were still in the lysosome while 40% resided in endoplasmic reticulum-labelled vacuoles (Figure 4). Further, there were more rod-shaped (undegraded) organisms in the absence of caspase-7 (Figure S5). Therefore, the restriction of infection in macrophages is achieved at least in part, by the delivery of more organisms to the lysosome when caspase-7 is activated.

The presumptive role of caspase-7 as an executioner caspase, involved in the cleavage of apoptotic substrates, is based primarily on its close relationship with caspase-3. However, recent reports demonstrate that caspase-7 and caspase-3 are functionally distinct [46],[55]. As reported by others, we found that unlike caspase7, caspase-3 was activated by *L. pneumophila* in A/J-derived macrophages and not in wild-type macrophages (Figure 2D). Therefore, our data demonstrate that caspase-3 and caspase-7 are not simultaneously activated during *L. pneumophila* infection as they are during apoptosis.

Furthermore, the absence of caspase-7 but not caspase-3 was accompanied with permissiveness to *L. pneumophila* infection *in vitro* and *in vivo* (Figure 3). Caspase-3 is also activated independently of the classical apoptosis pathways [55],[56], and reports have suggested that caspase-3 activation promotes cell survival [57]. Several studies suggest that caspase-3 activation is essential for establishment of *L. pneumophila* infection by mediating the cleavage of Rabaptin5 [55],[56]. These data strongly suggest different roles of caspase-7 versus caspase-3, as they restrict and permit *L. pneumophila* infection, respectively (Table S1).

Interestingly, TUNEL analysis of infected macrophage population revealed that during the first 2 hrs of infection, 20% of infected wild-type macrophages were apoptotic which may hinder the optimal replication of the organism, whereas infected macrophages lacking caspase-7, -1 or a functional Naip5 did not undergo early apoptosis (Figure 5F). These data agree with previous reports that suggest that *L. pneumophila* delay apoptosis in permissive macrophages to allow for the establishment of the replicative vacuole [46],[58]. Since early apoptosis is delayed in macrophages lacking caspase-1, caspase-7, or functional Naip5, and since these cell types are defective in caspase-7 activation in response to *L. pneumophila*, we conclude that caspase-7 activation contributes to early apoptosis observed in wild-type macrophages. However, our conclusion does not rule out the contribution of other mechanisms [55],[59],[60].

High MOI led to significant apoptosis in wild-type, caspase-7^−/−^ and A/J-derived macrophages but not in caspase-1^−/−^ cells (Figure 5C). Hence, caspase-1 appears as a more general regulator of *L. pneumophila*-triggered apoptosis. Caspase-7, however, regulated apoptosis only in the early stage of infection at low MOI. Similarly, Korfali *et al* showed that caspase-7 is involved earlier than other effector caspases in the apoptotic execution process in DT40 B lymphocytes [61]. Despite this, *L. pneumophila* replicated to a similar extent in macrophages lacking caspase-1 or caspase-7 (Figure 3), suggesting that for *L. pneumophila* to establish infection, it is particularly important to delay apoptosis during the early stages of infection.

Lungs from infected mice did not show significant differences in apoptosis after 3 days whether they lacked caspase-1, caspase-7, or a functional Naip5 (Figure S8). This does not exclude the role of cell death *in vivo* but strongly suggests that there must be at least one other complementary mechanism employed through caspase-7 to restrict infection.

Macrophages harbouring A/J Naip5 allele are capable of caspase-1 activation in response to *L. pneumophila* as reported by our group and by Lightfield *et al* [28],[29],[33], nevertheless, they are defective in caspase-7 activation (Figure 2E). Therefore, it is possible that the lack of caspase-7 activation is responsible at least in part, for the permissiveness of the A/J cells to *L. pneumophila* infection. Increasing evidence suggest that the Naip family of proteins may have yet uncharacterized functions [33], [62]–[64]. We propose that wild-type Naip5 mediates the activation of caspase-7 by caspase-1 during infections at low MOI. Our suggestion is supported by the fact that Naip5 interacts with Nlrc4 (which binds caspase-1) and with caspase-7 [31],[48],[65].

However, recent reports showed that the complete absence of Naip5 prevents caspase-1 activation [33]. The authors suggested that Naip5 is upstream of caspase-1, but it is possible that that Naip5 is required for proper assembly of the Nlrc4 inflammasome and hence caspase-1 activation but that A/J-derived Naip5 is partially functional as suggested by the authors [33]. This partial functionality may permit inflammasome assembly and caspase-1 activation but fail to mediate downstream caspase-7 activation, as suggested by our results. The isolation of the Nlrc4 inflammasome and identification of its components could clarify these possibilities.

Since IL-1β and IL-18 are downstream of caspase-1 and IL-1β has been implicated in controlling the maturation of the phagosome containing the Mycobacteria *zmp-1* mutant, we examined the role of IL-1β and IL-18 in *L. pneumophila* infection and in caspase-7 activation [66],[67]. IL-1β/IL-18 double knockout macrophages restricted *L. pneumophila* infection as efficiently as wild-type macrophages (Figure S6B). In addition, caspase-7 activity in the double-knockout cells was equal to that in wild-type cells (Figure S6A). Finally, exogenously-added IL-1β or antibodies against the corresponding receptors did not alter the number of *L. pneumophila* as measured by colony-forming-unit assays (Figure S6C). Therefore, our model suggests that caspase-7 activation and *L. pneumophila* restriction are mediated downstream of caspase-1 but independently of IL-1β and IL-18 (Table S1).

In summary, caspase-7 activation restricts *L. pneumophila* infection by mediating macrophage apoptosis during early stages of infection and by affecting the trafficking of the organism within the cell. How caspase-7 mediates these distinct functions remains unclear. It is possible that caspase-7 modulates host or bacterial factors involved in controlling apoptosis and vesicular trafficking in the cell. The identification of caspase-7 substrates during *L. pneumophila* infection is the focus of ongoing work.

Therefore, our findings establish a previously uncharacterized caspase-7 activation pathway downstream of the Nlrc4 inflammasome during *L. pneumophila* infection. Moreover, these results demonstrate a novel biological role for caspase-7 in the control of *L. pneumophila* intracellular infection in macrophages and in mice. This new pathway can be a target for compounds that could have therapeutic application in the context of intracellular infection.

## Materials and Methods

### Bacterial strains

*Legionella pneumophila* (*L. pneumophila*) strain Lp02, is a thymine auxotrophic derivative of Philadelphia-1 [19]. The *dotA* mutant strain Lp03 is defective in the Dot/Icm Type IV secretion system [68]. The *flaA* mutant *L. pneumophila* was previously described [69]. Bacterial strains were supplemented with a plasmid that complements thymine auxotrophy and expresses green fluorescent protein (GFP) at the post-exponential phase (PE) [22],[45]. *L. pneumophila* was cultured as described previously [22],[45] in ACES-yeast extract broth supplemented with ferric nitrate and L-cysteine. All experiments were performed in the absence of ferric nitrate and L-cysteine from the macrophage culture medium, to allow *L. pneumophila* multiplication only intracellularly. All *in vitro* infections were performed at an MOI of 0.5 for 30 minutes followed by rinsing of the infected macrophages which allowed the infection of 20–25% of macrophages with usually 1 organism, unless stated otherwise [28]. The quantification of the colony-forming units (CFU) *in vitro* and *in vivo* was performed as described [28].

### Mice

Wild-type C57BL/6 (B6), caspase-7^−/−^, caspase-3^−/−^, and A/J mice were previously described and purchased from the Jackson laboratory [41],[70],[71]. Caspase-1^−/−^ mice were from Dr. Amy Hise at Case Western University. MyD88^−/−^, TRIF^−/−^, and Nlrc4^−/−^ mice were previously described [72],[73]. All knockout mice were in C57BL/6 background. Caspase-1^−/−^ and caspase-7^−/−^ mice were backcrossed to C57BL/6 background for 10 generations and previously described [37],[70]. IL-1β/IL-18 double knockout mice [74] in C57BL/6 background were obtained from Dr. A. Zynchlinsky, Max-Plank-Institute, Berlin (authorized by Dr. S. Akira, Japan).

### Macrophages

Bone marrow macrophages were prepared from femurs of five to eight-week-old mice as previously described [28],[75].

### Plasmids and transfection

Mouse caspase-7 plasmid pCAGGS vector (LMBP 3818) was obtained from Gent University in Belgium. The plasmid was deposited by Dr. P. Vandenabeele [76]. Control plasmids used for transfection were obtained from Amaxa (pMaxGFP) or cloned in Dr. Wewer's Laboratory (pLenti-dsRed). To deliver control and caspase-7 plasmids into mouse macrophages, two approaches were used: transfection with SuperFect (Qiagen) and nucleofection with Amaxa (Lonza). Briefly, in transfection, mouse macrophages were seeded in 24-well plate at a density 0.5×10^6^ cells per well 24 hours prior the transfection. Next day, the media was changed leaving 400 µl of full media per well. To make a transfection mix, control or caspase-7 plasmids (1 µg/well) were mixed with SuperFect reagent (2.5 µl/well) in a total volume of 100 µl of serum-free media for 20 minutes. Then, transfection mix was added to the mouse macrophages bringing volume to 0.5 ml per well. Three hours later, transfection mix was replaced with 1 ml of full media and cells were left to recover for additional 24 hr before adding bacteria. In nucleofection approach, 6×10^6^ cells were resuspended in 100 µl of nucleofection solution (mouse macrophage nucleofector kit) supplemented with 10 µg plasmid. Plasmids were delivered into macrophages with Y-01 program. After nucleofection, macrophages were resuspended in 6 ml of full media and 0.5×10^6^ cells were plated per well in 24-well plate. Cell death was monitored throughout the assays. Bacteria were added 24 hr later.

### Fluorescence microscopy

Immunofluorescence experiments were performed as previously described [28]. *L. pneumophila* was detected with mouse anti-*Legionella* (Abcam) and secondary Texas Red conjugated antibody. Localization of markers on *L. pneumophila* phagosomes was performed as previously described [22]. Antibodies used were rat anti-lysosomal-associated membrane protein 1 (LAMP1; 1D4B; Developmental Hybridoma Bank) [77], rabbit anti-calreticulin (Stressgen Bioreagents) followed by fluorescent secondary antibodies (Molecular Probes). Nuclei were stained with nucleic acid dye 4′,6′-diamino-2-phenylindole (DAPI; Molecular Probes). In each experiment one hundred bacteria were scored. Experiments were performed at least three times. Samples were analyzed with The Zeiss 510 META Laser Scanning Confocal microscope and Zeiss Axioplan 2 upright microscope at The Ohio State University Microscopy Core Facility.

### *L. pneumophila* growth in macrophages

Macrophages were plated at 5×10^5^ cells per well and infected as described above [28]. At designated time points, macrophages were lysed and plated on AYE plates for colony forming units (CFUs). When indicated, macrophages were treated with recombinant IL-1β or IL-18 (Calbiochem) or with anti-mIL-1-RI or mIL-18 R/IL-1 R5 antibodies (R & D systems) at time of infection. Caspase-8 inhibitor (Z-IETD-FMK), caspase-9 inhibitor (Z-LEHD-FMK), or caspase-1 inhibitor (Z-YVAD-FMK) (Calbiochem) were used at 50 µM concentration when indicated. Inhibitors were maintained during the course of infection.

### Immunoblotting

Cell extracts were prepared and immunoblotted with an anti-body that recognizes the large subunit of mouse caspase-1, -3 or -7 (Cell Signalling), followed by appropriate secondary anti-rabbit antibody as described [28]. When indicated, macrophages were permeabilized with 10 ng/ml streptolysin O for 5 minutes in the absence or presence of ligands as previously described [28],[39], then rinsed and incubated for 2 hours [28]. Purified bacterial ligands were purchased from Invivogen.

### Cytokine measurements

IL-1β measurements were performed as previously described [72],[73]. Experiments were performed in triplicates.

### Cytotoxicity assays

The percentage of macrophage death was determined by measuring the release of host cell cytoplasmic LDH using the CytoTox 96 non-radioactive cytotoxicity assay (Promega) as previously described [28],[45]. The apoptosis inducer doxorubicin was added at 100 ng/ml, when indicated (Calbiochem). *In vitro* quantification of cytoplasmic (apoptosis) and extracellular (necrosis) histone-associated DNA fragments was performed using The Cell Death Detection ELISA^plus^ photometric enzyme immunoassay kit from Roche to the specifications of the manufacturer. Apotosis of macrophages *in vitro* was assessed with fluorescent TUNEL (terminal deoxynucleotidyl transferase-mediated dUTP nick end-labelling) assay according to the manufacturer's specifications using *In Situ* Cell Death Detection Kit, TMR red from Roche. Experiments were performed in triplicates. Sections of infected mice lungs were stained for apoptosis using Apop Tag In Situ Apoptosis Detection Kit (Chemicon). TUNEL-positive stained cells (brown) were evaluated by capturing digital images using a 20× objective lens and covering the entire lung (at least 32 images per lung). All samples were handled in a blinded manner. The percent of brown pixels per high powered field (HPF) were quantified using Adobe Photoshop CS2 software histogram analysis.

### Mouse *in vivo* Infection

Wild-type C57BL/6 and caspase-7^−/−^ mice were infected intra-tracheally with 1×10^6^ wild-type bacteria, and the number of bacteria in the lungs was determined at 6 hours and at 96 hours post-infection [28],[75]. All animal experiments performed were done according to animal protocols approved by the Animal Care Use Committee of The Ohio State University College of Medicine.

### Statistical analysis

All experiments were done at least three independent times and yielded similar results. The data points represent the average ±S.D. Data were analyzed by Student's t-test. *, *P* value≤0.05 and was considered significant.

## Supporting Information

Figure S1 Caspase-7 activation by *L. pneumophila* at high multiplicity of infection (MOI) is independent of caspase-1. (A) Wild-type C57BL/6 (B6) and caspase-7^−/−^ (casp-7^−/−^) derived macrophages were not treated (No treat) or infected with *L. pneumophila* (Leg). (B) Caspase-1^−/−^ (casp-1^−/−^) macrophages were not treated (No treat) or infected with Leg or the flagellin mutant (Fla). (C) B6 and casp-7^−/−^ macrophages were infected with Fla mutant at MOI of 0.5, 5 or 20. (A–C) Infections were at MOI of 0.5, 5, or 20 for 2 hrs, then cell lysates were analyzed by western blots with anti-caspase-7 antibodies.
(0.05 MB PDF)

Figure S2 Caspase-7 activation by *L. pneumophila* does not require MyD88 or TRIF. B6 and MyD88^−/−^ (A), or TRIF^−/−^ (B) macrophages were infected with *L. pneumophila* (Leg) or with flagellin mutant (Fla) for 2 hrs then cell lysates were analyzed by western blot using anti-caspase-7 antibodies.
(0.05 MB PDF)

Figure S3 Caspase-7 activation by *L. pneumophila* is independent of caspase-8 or -9. (A) Wild-type C57BL/6 (B6) macrophages were infected with Leg in the presence or absence of 50 µM of caspase-8 inhibitor (IETD), caspase-9 inhibitor (LEHD), or caspase-1 inhibitor (YVAD). (B) B6 macrophages were treated with the apoptosis-inducing drug doxorubicin in the presence or absence of IETD, or LEHD or both. (A,B) Cell lysates were analyzed by western blots with anti-caspase-7 antibody. Densitometric analysis of the cleaved caspase-7 band is expressed relative to non-treated samples and the values are indicated immediately below the caspase-7 blots. The lower panels show actin blots as loading controls.
(0.05 MB PDF)

Figure S4 Transfection of macrophages lacking caspase-7 with caspase-7 plasmid restores restriction to *L. pneumophila* infection. (A) Caspase-7^−/−^ macrophages (Casp-7^−/−^) were transfected with plasmids expressing either caspase-7 (C7 PL) or the red fluorescent protein (RFP PL) using Superfect (SF) or Amaxa (Ax), then cell lysates were analyzed by western blots with anti-caspase-7 antibodies. (B) Casp-7^−/−^ macrophages transfected or not with C7 plasmid or RFP by SF were infected with *L. pneumophila* and colony forming units were quantified at designated time points. The results represent the mean of three independent experiments ±SD.
(0.04 MB PDF)

Figure S5 Differential trafficking of *L. pneumophila* in caspase-7^−/−^ macrophages requires a functional type IV secretion system. (A) Wild-type C57BL/6 (B6), caspase-7^−/−^ (casp-7^−/−^), and caspase-1^−/−^ (casp-1^−/−^) macrophages were seeded on cover slips and infected with the Dot/Icm type IV secretion mutant for 2 hrs and bacteria were examined for co-localization with LAMP-1. (B) B6 macrophages seeded over cover slips were infected with wild-type *L. pneumophila* then the organism and its degradation fragments were detected with anti-*Legionella* antibody and secondary fluorescent antibody. *, P value≤0.05. The results represent the mean of three independent experiments ±SD.
(0.01 MB PDF)

Figure S6 IL-1β and IL-18 do not affect caspase-7 activation or *L. pneumophila* infection. (A) Wild-type C57BL/6 (B6) and IL-1β^−/−^/IL-18^−/−^ derived macrophages were not treated (No treat) or infected with wild-type *L. pneumophila* (Leg) or flagellin mutant (Fla) then cell lysates were analysed by western blots with ani-caspase-7 antibodies. (B) B6, IL-1β^−/−^/IL-18^−/−^, Caspase-7^−/−^ (casp-7^−/−^), and A/J-derived (Naip5^AJ^) macrophages were infected with wild-type *L. pneumophila* and colony forming units (CFU) were recovered at designated time points. (C) B6 and casp-7^−/−^ macrophages were infected with *L. pneumophila* in the presence or absence of exogenous recombinant (r) IL-1β, rIL-18, IL-1β receptor (R) antibody (AB), or IL-18 R AB, then CFUs were scored at indicated time points. Data represent the mean of three independent experiments ±SD.
(0.03 MB PDF)

Figure S7 Caspase-7 does not regulate cytokine secretion in macrophages. (A) Macrophages from wild-type C57BL/6 (B6), caspase-1^−/−^ (casp-1^−/−^), or caspase-7^−/−^ (casp-7^−/−^) mice were not infected (white bars) or infected (black bars) with *L. pneumophila* (A) or *Salmonella typhimurium* (*Salmonella*) (B), then cell supernatants were examined for IL-1β release. Data represent the mean of three independent experiments ±SD. *, P value≤0.05.
(0.05 MB PDF)

Figure S8 *L. pneumophila* induces mild pulmonary apoptosis in wild-type mice and in mice lacking caspase-7, -1, or wild-type Naip5. (A) Lungs from infected wild-type C57BL/6, caspase-1^−/−^, caspase-7^−/−^, and A/J mice were harvested at 72 hrs post infection and stained for TUNEL to detect apoptotic nuclei. (B) TUNEL-positive stained cells (brown) were evaluated by capturing digital images and the percent of brown pixels per high powered field were quantified. Data represent the mean of at least 32 images per lung ±SD.
(0.09 MB PDF)

Figure S9 Human monocytes do not activate caspase-1 or caspase-7 in response to *L. pneumophila* infection. Fresh human monocytes were infected or not (No treat) with *L. pneumophila* (Leg) or *Salmonella* at an MOI of 0.5 for 2 hrs. Then, cell lysates were analyzed by western blots using anti-caspase-1 (A) and -caspase-7 (B) antibodies. Data are representative of three experiments from three independent donors.
(0.06 MB PDF)

Table S1 *L. pneumophila* replicates in the absence of caspase-7 activation in Nlrc4^−/−^, caspase-1^−/−^ (casp-1^−/−^), caspase-7^−/−^ (casp-7^−/−^), and in A/J (Naip5^AJ^) macrophages. Caspase-7 activation restricts *L. pneumophila* replication in C57BL/6 (B6) and in IL-1β^−/−^/IL-18^−/−^ macrophages.
(0.02 MB PDF)

## References

[1] Li J, Yuan J (2008). Caspases in apoptosis and beyond.. Oncogene.

[2] Martinon F, Tschopp J (2004). Inflammatory caspases: linking an intracellular innate immune system to autoinflammatory diseases.. Cell.

[3] Kuribayashi K, Mayes PA, El-Deiry WS (2006). What are caspases 3 and 7 doing upstream of the mitochondria?. Cancer Biology & Therapy.

[4] Stennicke HR, Salvesen GS (1997). Biochemical characteristics of caspases-3, -6, -7, and -8.. Journal of Biological Chemistry.

[5] Thornberry NA, Molineaux SM (1995). Interleukin-1 beta converting enzyme: a novel cysteine protease required for IL-1 beta production and implicated in programmed cell death.. Protein Science.

[6] Martinon F, Agostini L, Meylan E, Tschopp J (2004). Identification of bacterial muramyl dipeptide as activator of the NALP3/cryopyrin inflammasome.. Current Biology.

[7] Ting JP, Lovering RC, Alnemri ES, Bertin J, Boss JM (2008). The NLR gene family: a standard nomenclature.. Immunity.

[8] Ting JP, Willingham SB, Bergstralh DT (2008). NLRs at the intersection of cell death and immunity.. Nat Rev Immunol.

[9] Sutterwala FS, Ogura Y, Zamboni DS, Roy CR, Flavell RA (2006). NALP3: a key player in caspase-1 activation.. Journal of Endotoxin Research.

[10] Franchi L, Park JH, Shaw MH, Marina-Garcia N, Chen G (2008). Intracellular NOD-like receptors in innate immunity, infection and disease.. Cell Microbiol.

[11] Kanneganti TD, Lamkanfi M, Nunez G (2007). Intracellular NOD-like receptors in host defense and disease.. Immunity.

[12] Wilmanski JM, Petnicki-Ocwieja T, Kobayashi KS (2008). NLR proteins: integral members of innate immunity and mediators of inflammatory diseases.. J Leukoc Biol.

[13] Gavrilin MA, Bouakl IJ, Knatz NL, Duncan MD, Hall MW (2006). Internalization and phagosome escape required for Francisella to induce human monocyte IL-1beta processing and release.. Proc Natl Acad Sci U S A.

[14] Butchar JP, Cremer TJ, Clay CD, Gavrilin MA, Wewers MD (2008). Microarray analysis of human monocytes infected with *Francisella tularensis* identifies new targets of host response subversion.. PLoS ONE.

[15] Henry T, Monack DM (2007). Activation of the inflammasome upon *Francisella tularensis* infection: interplay of innate immune pathways and virulence factors.. Cell Microbiol.

[16] Monack DM (2008). The inflammasome: a key player in the inflammation triggered in response to bacterial pathogens.. J Pediatr Gastroenterol Nutr.

[17] Delbridge LM, O'Riordan MX (2007). Innate recognition of intracellular bacteria.. Curr Opin Immunol.

[18] Sarkar A, Hall MW, Exline M, Hart J, Knatz N (2006). Caspase-1 regulates *Escherichia coli* sepsis and splenic B cell apoptosis independently of interleukin-1beta and interleukin-18.. American Journal of Respiratory & Critical Care Medicine.

[19] McDade JE, Shepard CC, Fraser DW, Tsai TR, Redus MA (1977). Legionnaires' disease: isolation of a bacterium and demonstration of its role in other respiratory disease.. N Engl J Med.

[20] Horwitz MA (1983). Formation of a novel phagosome by the Legionnaires' disease bacterium (*Legionella pneumophila*) in human monocytes.. J Exp Med.

[21] Kagan JC, Roy CR (2002). *Legionella* phagosomes intercept vesicular traffic from endoplasmic reticulum exit sites.. Nat Cell Biol.

[22] Amer AO, Swanson MS (2005). Autophagy is an immediate macrophage response to *Legionella pneumophila*.. Cell Microbiol.

[23] Tilney LG, Harb OS, Connelly PS, Robinson CG, Roy CR (2001). How the parasitic bacterium *Legionella pneumophila* modifies its phagosome and transforms it into rough ER: implications for conversion of plasma membrane to the ER membrane.. Journal of Cell Science.

[24] Horwitz MA, Maxfield FR (1984). *Legionella pneumophila* inhibits acidification of its phagosome in human monocytes.. Journal of Cell Biology.

[25] Molmeret M, Bitar DM, Han L, Kwaik YA (2004). Cell biology of the intracellular infection by *Legionella pneumophila*.. Microbes & Infection.

[26] Roy CR, Tilney LG (2002). The road less traveled: transport of *Legionella* to the endoplasmic reticulum.. J Cell Biol.

[27] Isberg RR, O'Connor TJ, Heidtman M (2009). The *Legionella pneumophila* replication vacuole: making a cosy niche inside host cells.. Nat Rev Microbiol.

[28] Amer A, Franchi L, Kanneganti TD, Body-Malapel M, Ozoren N (2006). Regulation of *Legionella* phagosome maturation and infection through flagellin and host Ipaf.. J Biol Chem.

[29] Lamkanfi M, Amer A, Kanneganti TD, Munoz-Planillo R, Chen G (2007). The Nod-like receptor family member Naip5/Birc1e restricts *Legionella pneumophila* growth independently of caspase-1 activation.. J Immunol.

[30] Lamkanfi M, Kanneganti TD, Franchi L, Nunez G (2007). Caspase-1 inflammasomes in infection and inflammation.. Journal of Leukocyte Biology.

[31] Zamboni DS, Kobayashi KS, Kohlsdorf T, Ogura Y, Long EM (2006). The Birc1e cytosolic pattern-recognition receptor contributes to the detection and control of *Legionella pneumophila* infection.. Nat Immunol.

[32] Losick VP, Stephan K, Smirnova II, Isberg RR, Poltorak A (2009). A hemidominant Naip5 allele in mouse strain MOLF/Ei-derived macrophages restricts *Legionella pneumophila* intracellular growth.. Infect Immun.

[33] Lightfield KL, Persson J, Brubaker SW, Witte CE, von Moltke J (2008). Critical function for Naip5 in inflammasome activation by a conserved carboxy-terminal domain of flagellin.. Nat Immunol.

[34] Wright EK, Goodart SA, Growney JD, Hadinoto V, Endrizzi MG (2003). Naip5 affects host susceptibility to the intracellular pathogen *Legionella pneumophila*.. Curr Biol.

[35] Diez E, Lee SH, Gauthier S, Yaraghi Z, Tremblay M (2003). Birc1e is the gene within the Lgn1 locus associated with resistance to *Legionella pneumophila*.. Nature Genetics.

[36] Yamamoto Y, Klein TW, Newton CA, Widen R, Friedman H (1988). Growth of *Legionella pneumophila* in thioglycolate-elicited peritoneal macrophages from A/J mice.. Infect Immun.

[37] Lamkanfi M, Kanneganti TD, Van Damme P, Vanden Berghe T, Vanoverberghe I (2008). Targeted peptidecentric proteomics reveals caspase-7 as a substrate of the caspase-1 inflammasomes.. Mol Cell Proteomics.

[38] Van de Craen M, Declercq W, Van den brande I, Fiers W, Vandenabeele P (1999). The proteolytic procaspase activation network: an in vitro analysis.. Cell Death & Differentiation.

[39] Walev I, Bhakdi SC, Hofmann F, Djonder N, Valeva A (2001). Delivery of proteins into living cells by reversible membrane permeabilization with streptolysin-O.. Proc Natl Acad Sci U S A.

[40] Fortier A, Diez E, Gros P (2005). Naip5/Birc1e and susceptibility to *Legionella pneumophila*.. Trends Microbiol.

[41] Dietrich WF, Damron DM, Isberg RR, Lander ES, Swanson MS (1995). *Lgn1*, a gene that determines susceptibility to *Legionella pneumophila*, maps to mouse chromosome 13.. Genomics.

[42] Fortin A, Diez E, Rochefort D, Laroche L, Malo D (2001). Recombinant congenic strains derived from A/J and C57BL/6J: a tool for genetic dissection of complex traits.. Genomics.

[43] Amer AO, Swanson MS (2002). A phagosome of one's own: a microbial guide to life in the macrophage.. Current Opinion in Microbiology.

[44] Derre I, Isberg RR (2004). Macrophages from mice with the restrictive Lgn1 allele exhibit multifactorial resistance to *Legionella pneumophila*.. Infect Immun.

[45] Sturgill-Koszycki S, Swanson MS (2000). *Legionella pneumophila* replication vacuoles mature into acidic, endocytic organelles.. J Exp Med.

[46] Abu-Zant A, Santic M, Molmeret M, Jones S, Helbig J (2005). Incomplete activation of macrophage apoptosis during intracellular replication of *Legionella pneumophila*.. Infection & Immunity.

[47] Horwitz MA (1983). The Legionnaires' disease bacterium (*Legionella pneumophila*) inhibits phagosome-lysosome fusion in human monocytes.. J Exp Med.

[48] Vinzing M, Eitel J, Lippmann J, Hocke AC, Zahlten J (2008). NAIP and Ipaf control *Legionella pneumophila* replication in human cells.. J Immunol.

[49] Walsh JG, Cullen SP, Sheridan C, Luthi AU, Gerner C (2008). Executioner caspase-3 and caspase-7 are functionally distinct proteases.. Proc Natl Acad Sci U S A.

[50] Kinchen JM, Ravichandran KS (2008). Phagosome maturation: going through the acid test.. Nat Rev Mol Cell Biol.

[51] Alonso A, Garcia-del Portillo F (2004). Hijacking of eukaryotic functions by intracellular bacterial pathogens.. Int Microbiol.

[52] Scott CC, Botelho RJ, Grinstein S (2003). Phagosome maturation: a few bugs in the system.. J Membr Biol.

[53] Wiater LA, Dunn K, Maxfield FR, Shuman HA (1998). Early events in phagosome establishment are required for intracellular survival of *Legionella pneumophila.*. Infect Immun.

[54] Segal G, Shuman HA (1998). How is the intracellular fate of the Legionella pneumophila phagosome determined?. Trends in Microbiology.

[55] Molmeret M, Zink SD, Han L, Abu-Zant A, Asari R (2004). Activation of caspase-3 by the Dot/Icm virulence system is essential for arrested biogenesis of the *Legionella*-containing phagosome.. Cell Microbiol.

[56] Zink SD, Pedersen L, Cianciotto NP, Abu-Kwaik Y (2002). The Dot/Icm type IV secretion system of *Legionella pneumophila* is essential for the induction of apoptosis in human macrophages.. Infect Immun.

[57] Cheng C, Zochodne DW (2003). Sensory neurons with activated caspase-3 survive long-term experimental diabetes.. Diabetes.

[58] Banga S, Gao P, Shen X, Fiscus V, Zong WX (2007). *Legionella pneumophila* inhibits macrophage apoptosis by targeting pro-death members of the Bcl2 protein family.. Proc Natl Acad Sci U S A.

[59] Shin S, Case CL, Archer KA, Nogueira CV, Kobayashi KS (2008). Type IV secretion-dependent activation of host MAP kinases induces an increased proinflammatory cytokine response to *Legionella pneumophila*.. PLoS Pathog.

[60] Abu-Zant A, Jones S, Asare R, Suttles J, Price C (2007). Anti-apoptotic signalling by the Dot/Icm secretion system of *L. pneumophila*.. Cell Microbiol.

[61] Korfali N, Ruchaud S, Loegering D, Bernard D, Dingwall C (2004). Caspase-7 gene disruption reveals an involvement of the enzyme during the early stages of apoptosis.. Journal of Biological Chemistry.

[62] Rajalingam K, Dikic I (2009). Inhibitors of apoptosis catch ubiquitin.. Biochem J.

[63] Vince JE, Wong WW, Khan N, Feltham R, Chau D (2007). IAP antagonists target cIAP1 to induce TNFalpha-dependent apoptosis.. Cell.

[64] Varfolomeev E, Blankenship JW, Wayson SM, Fedorova AV, Kayagaki N (2007). IAP antagonists induce autoubiquitination of c-IAPs, NF-kappaB activation, and TNFalpha-dependent apoptosis.. Cell.

[65] Maier JK, Lahoua Z, Gendron NH, Fetni R, Johnston A (2002). The neuronal apoptosis inhibitory protein is a direct inhibitor of caspases 3 and 7.. J Neurosci.

[66] Lazarevic V, Martinon F (2008). Linking inflammasome activation and phagosome maturation.. Cell Host Microbe.

[67] Master SS, Rampini SK, Davis AS, Keller C, Ehlers S (2008). Mycobacterium tuberculosis prevents inflammasome activation.. Cell Host Microbe.

[68] Berger KH, Merriam JJ, Isberg RR (1994). Altered intracellular targeting properties associated with mutations in the *Legionella pneumophila* dotA gene.. Mol Microbiol.

[69] Molofsky AB, Shetron-Rama LM, Swanson MS (2005). Components of the *Legionella pneumophila* flagellar regulon contribute to multiple virulence traits, including lysosome avoidance and macrophage death.. Infect Immun.

[70] Lakhani SA, Masud A, Kuida K, Porter GA, Booth CJ (2006). Caspases 3 and 7: key mediators of mitochondrial events of apoptosis.[see comment].. Science.

[71] Franchi L, Amer A, Body-Malapel M, Kanneganti TD, Ozoren N (2006). Cytosolic flagellin requires Ipaf for activation of caspase-1 and interleukin 1beta in salmonella-infected macrophages.[see comment].. Nature Immunology.

[72] Kanneganti TD, Body-Malapel M, Amer A, Park JH, Whitfield J (2006). Critical role for Cryopyrin/Nalp3 in activation of caspase-1 in response to viral infection and double-stranded RNA.. Journal of Biological Chemistry.

[73] Kanneganti TD, Ozoren N, Body-Malapel M, Amer A, Park JH (2006). Bacterial RNA and small antiviral compounds activate caspase-1 through cryopyrin/Nalp3.. Nature.

[74] Kuida K, Lippke JA, Ku G, Harding MW, Livingston DJ (1995). Altered cytokine export and apoptosis in mice deficient in interleukin-1 beta converting enzyme.. Science.

[75] Brieland J, Freeman P, Kunkel R, Chrisp C, Hurley M (1994). Replicative *Legionella pneumophila* lung infection in intratracheally inoculated A/J mice. A murine model of human Legionnaires' disease.. Am J Pathol.

[76] Van de Craen M, Vandenabeele P, Declercq W, Van den Brande I, Van Loo G (1997). Characterization of seven murine caspase family members.. FEBS Lett.

[77] Huynh KK, Eskelinen EL, Scott CC, Malevanets A, Saftig P (2007). LAMP proteins are required for fusion of lysosomes with phagosomes.. EMBO Journal.

