# Supplementary figures and images for: Caspase-7 Activation by the Nlrc4/Ipaf Inflammasome Restricts Legionella pneumophila Infection
Source: PLoS Pathog. 2009 Apr 3;5(4):e1000361. doi: 10.1371/journal.ppat.1000361 (PMC2657210; doi:10.1371/journal.ppat.1000361)

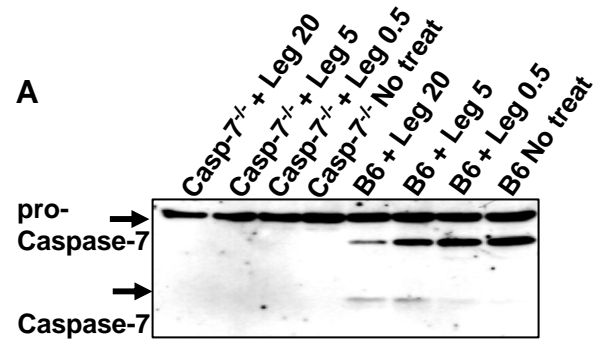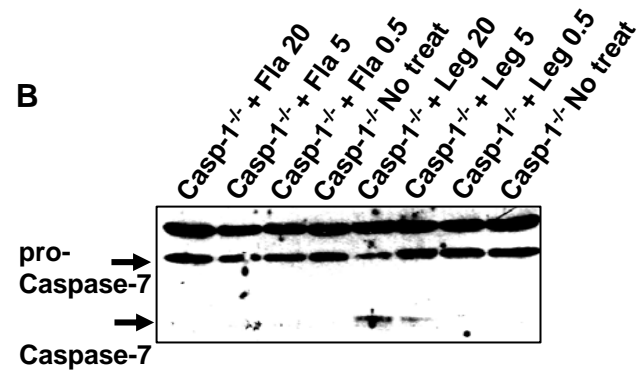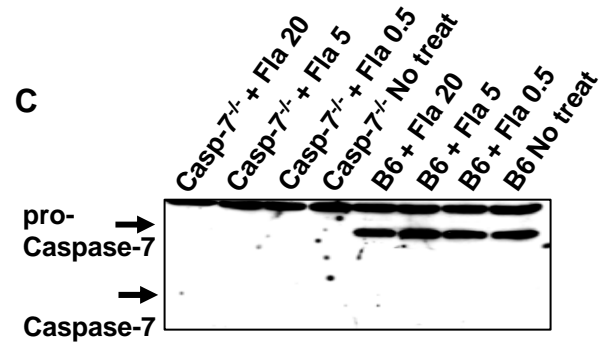

Supplement: Figure S1 — Caspase-7 activation by L. pneumophila at high multiplicity of infection (MOI) is independent of caspase-1. (A) Wild-type C57BL/6 (B6) and caspase-7−/− (casp-7−/−) derived macrophages were not treated (No treat) or infected with L. pneumophila (Leg). (B) Caspase-1−/− (casp-1−/−) macrophages were not treated (No treat) or infected with Leg or the flagellin mutant (Fla). (C) B6 and casp-7−/− macrophages were infected with Fla mutant at MOI of 0.5, 5 or 20. (A–C) Infections were at MOI of 0.5, 5, or 20 for 2 hrs, then cell lysates were analyzed by western blots with anti-caspase-7 antibodies. (0.05 MB PDF) [file ppat.1000361.s001.pdf]

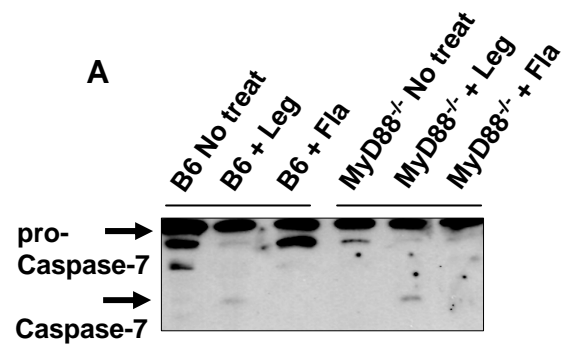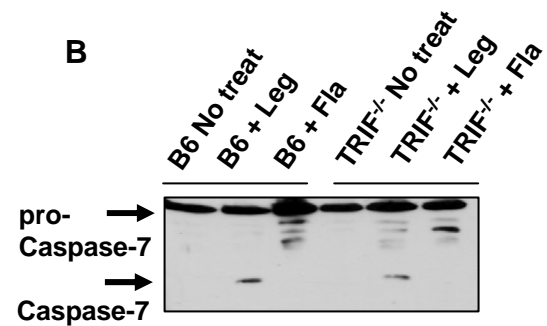

Supplement: Figure S2 — Caspase-7 activation by L. pneumophila does not require MyD88 or TRIF. B6 and MyD88−/− (A), or TRIF−/− (B) macrophages were infected with L. pneumophila (Leg) or with flagellin mutant (Fla) for 2 hrs then cell lysates were analyzed by western blot using anti-caspase-7 antibodies. (0.05 MB PDF) [file ppat.1000361.s002.pdf]

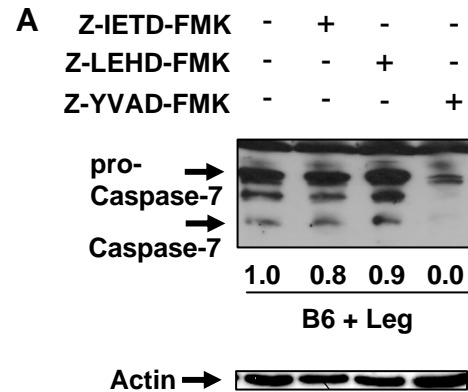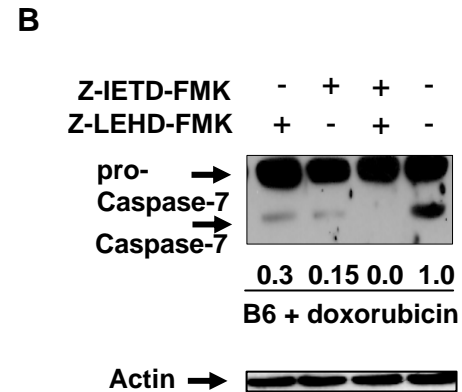

Supplement: Figure S3 — Caspase-7 activation by L. pneumophila is independent of caspase-8 or -9. (A) Wild-type C57BL/6 (B6) macrophages were infected with Leg in the presence or absence of 50 µM of caspase-8 inhibitor (IETD), caspase-9 inhibitor (LEHD), or caspase-1 inhibitor (YVAD). (B) B6 macrophages were treated with the apoptosis-inducing drug doxorubicin in the presence or absence of IETD, or LEHD or both. (A,B) Cell lysates were analyzed by western blots with anti-caspase-7 antibody. Densitometric analysis of the cleaved caspase-7 band is expressed relative to non-treated samples and the values are indicated immediately below the caspase-7 blots. The lower panels show actin blots as loading controls. (0.05 MB PDF) [file ppat.1000361.s003.pdf]

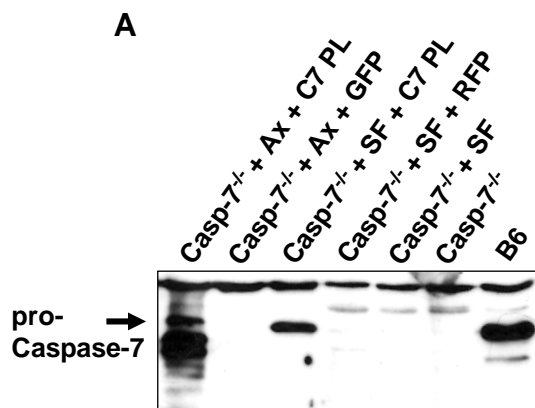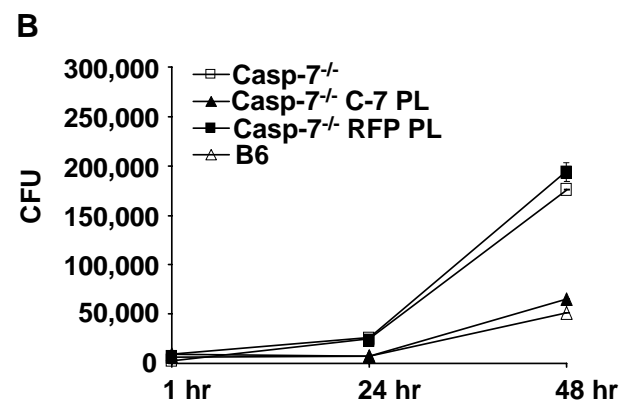

Supplement: Figure S4 — Transfection of macrophages lacking caspase-7 with caspase-7 plasmid restores restriction to L. pneumophila infection. (A) Caspase-7−/− macrophages (Casp-7−/−) were transfected with plasmids expressing either caspase-7 (C7 PL) or the red fluorescent protein (RFP PL) using Superfect (SF) or Amaxa (Ax), then cell lysates were analyzed by western blots with anti-caspase-7 antibodies. (B) Casp-7−/− macrophages transfected or not with C7 plasmid or RFP by SF were infected with L. pneumophila and colony forming units were quantified at designated time points. The results represent the mean of three independent experiments ±SD. (0.04 MB PDF) [file ppat.1000361.s004.pdf]

**A**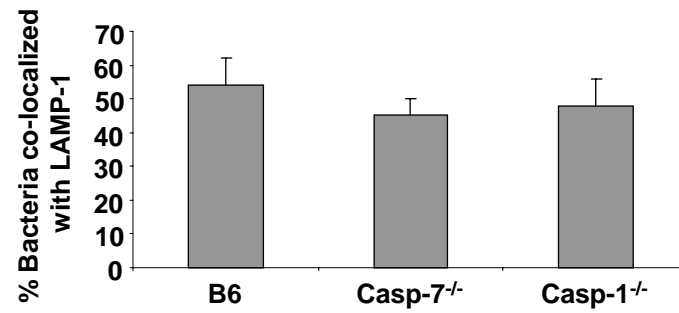**B**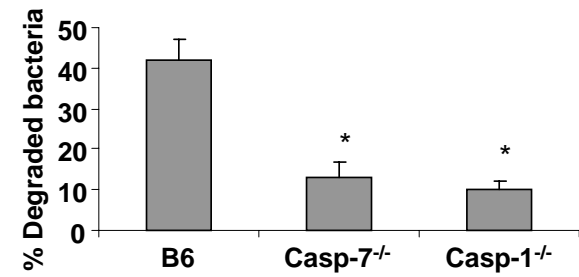

Supplement: Figure S5 — Differential trafficking of L. pneumophila in caspase-7−/− macrophages requires a functional type IV secretion system. (A) Wild-type C57BL/6 (B6), caspase-7−/− (casp-7−/−), and caspase-1−/− (casp-1−/−) macrophages were seeded on cover slips and infected with the Dot/Icm type IV secretion mutant for 2 hrs and bacteria were examined for co-localization with LAMP-1. (B) B6 macrophages seeded over cover slips were infected with wild-type L. pneumophila then the organism and its degradation fragments were detected with anti-Legionella antibody and secondary fluorescent antibody. *, P value≤0.05. The results represent the mean of three independent experiments ±SD. (0.01 MB PDF) [file ppat.1000361.s005.pdf]

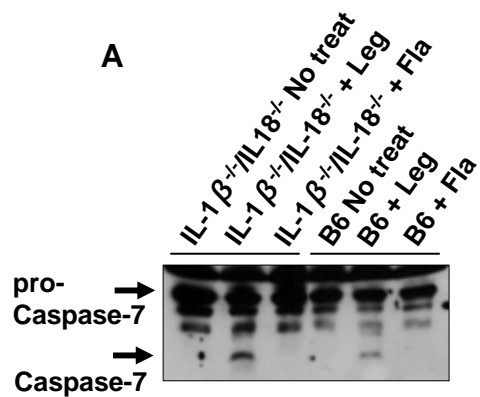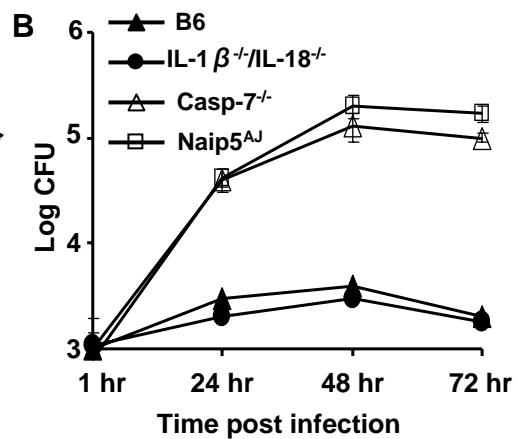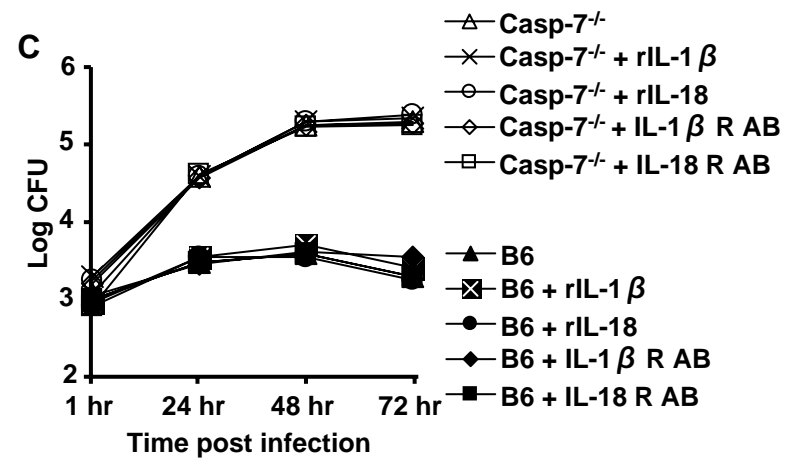

Supplement: Figure S6 — IL-1β and IL-18 do not affect caspase-7 activation or L. pneumophila infection. (A) Wild-type C57BL/6 (B6) and IL-1β−/−/IL-18−/− derived macrophages were not treated (No treat) or infected with wild-type L. pneumophila (Leg) or flagellin mutant (Fla) then cell lysates were analysed by western blots with ani-caspase-7 antibodies. (B) B6, IL-1β−/−/IL-18−/−, Caspase-7−/− (casp-7−/−), and A/J-derived (Naip5AJ) macrophages were infected with wild-type L. pneumophila and colony forming units (CFU) were recovered at designated time points. (C) B6 and casp-7−/− macrophages were infected with L. pneumophila in the presence or absence of exogenous recombinant (r) IL-1β, rIL-18, IL-1β receptor (R) antibody (AB), or IL-18 R AB, then CFUs were scored at indicated time points. Data represent the mean of three independent experiments ±SD. (0.03 MB PDF) [file ppat.1000361.s006.pdf]

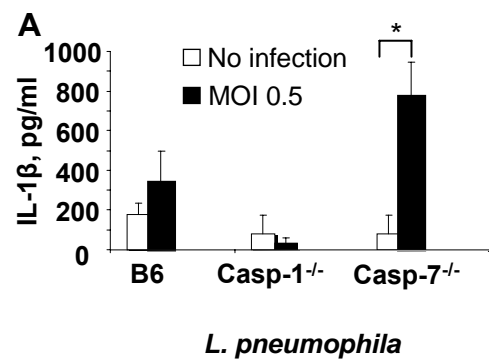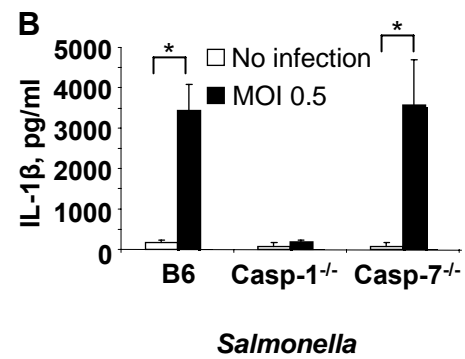

Supplement: Figure S7 — Caspase-7 does not regulate cytokine secretion in macrophages. (A) Macrophages from wild-type C57BL/6 (B6), caspase-1−/− (casp-1−/−), or caspase-7−/− (casp-7−/−) mice were not infected (white bars) or infected (black bars) with L. pneumophila (A) or Salmonella typhimurium (Salmonella) (B), then cell supernatants were examined for IL-1β release. Data represent the mean of three independent experiments ±SD. *, P value≤0.05. (0.05 MB PDF) [file ppat.1000361.s007.pdf]

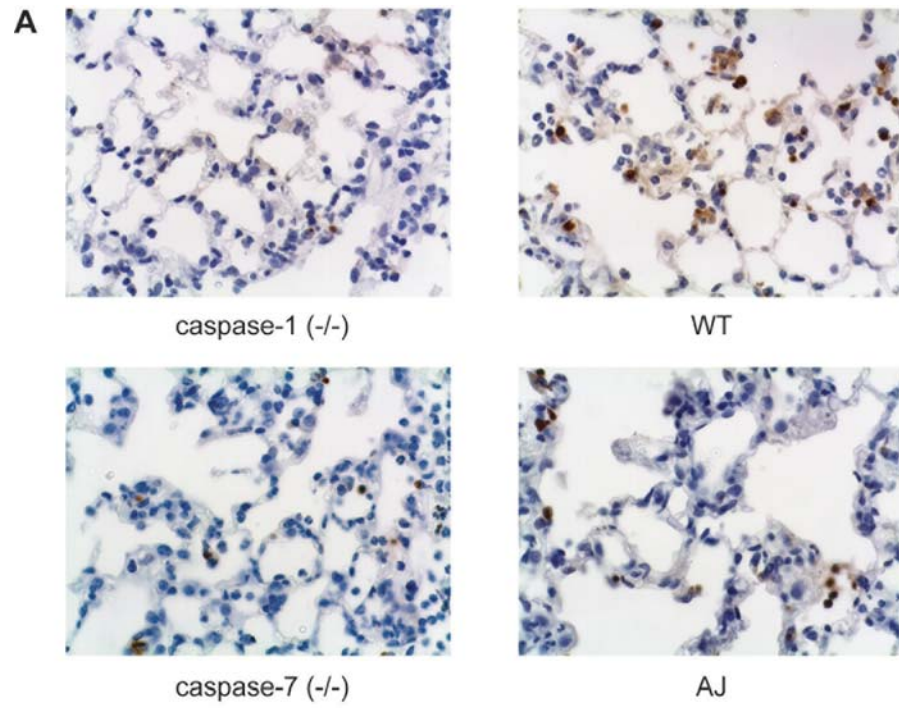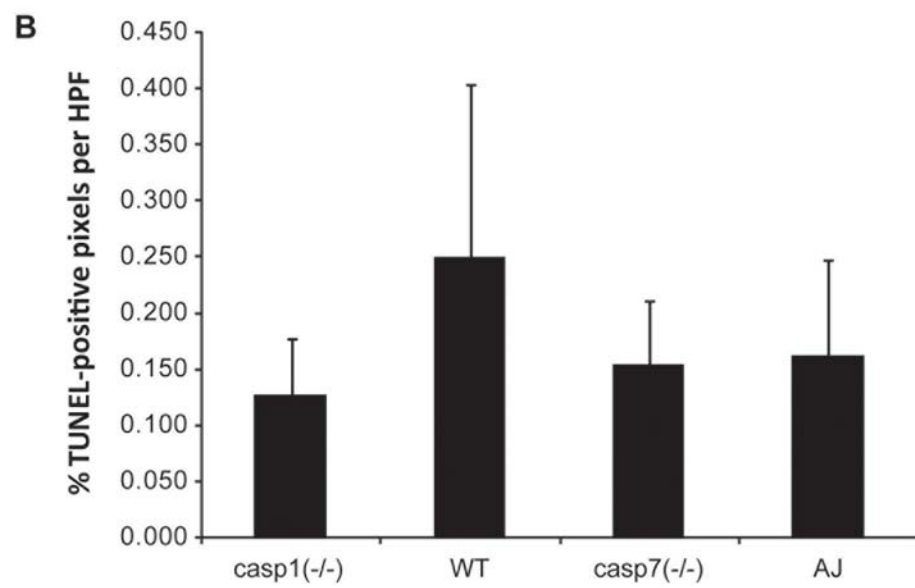

Supplement: Figure S8 — L. pneumophila induces mild pulmonary apoptosis in wild-type mice and in mice lacking caspase-7, -1, or wild-type Naip5. (A) Lungs from infected wild-type C57BL/6, caspase-1−/−, caspase-7−/−, and A/J mice were harvested at 72 hrs post infection and stained for TUNEL to detect apoptotic nuclei. (B) TUNEL-positive stained cells (brown) were evaluated by capturing digital images and the percent of brown pixels per high powered field were quantified. Data represent the mean of at least 32 images per lung ±SD. (0.09 MB PDF) [file ppat.1000361.s008.pdf]

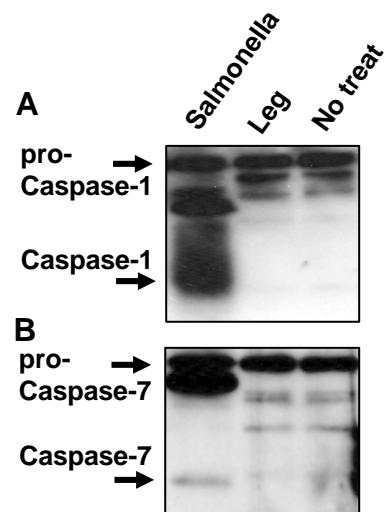

Supplement: Figure S9 — Human monocytes do not activate caspase-1 or caspase-7 in response to L. pneumophila infection. Fresh human monocytes were infected or not (No treat) with L. pneumophila (Leg) or Salmonella at an MOI of 0.5 for 2 hrs. Then, cell lysates were analyzed by western blots using anti-caspase-1 (A) and -caspase-7 (B) antibodies. Data are representative of three experiments from three independent donors. (0.06 MB PDF) [file ppat.1000361.s009.pdf]
