# Supplementary material for: Caspase-7 Activation by the Nlrc4/Ipaf Inflammasome Restricts Legionella pneumophila Infection
Source: PLoS Pathog. 2009 Apr 3;5(4):e1000361. doi: 10.1371/journal.ppat.1000361 (PMC2657210; doi:10.1371/journal.ppat.1000361)
Supplement: Table S1 — L. pneumophila replicates in the absence of caspase-7 activation in Nlrc4−/−, caspase-1−/− (casp-1−/−), caspase-7−/− (casp-7−/−), and in A/J (Naip5AJ) macrophages. Caspase-7 activation restricts L. pneumophila replication in C57BL/6 (B6) and in IL-1β−/−/IL-18−/− macrophages. (0.02 MB PDF) [file ppat.1000361.s010.pdf]

Mice strain	B6	$Nlrc4^{-/-}$	$Casp\text{-}1^{-/-}$	$Casp\text{-}3^{-/-}$	$Casp\text{-}7^{-/-}$	$Naip5^{AJ}$	$IL\text{-}1\beta^{-/-}$/ $IL\text{-}18^{-/-}$
Casp-1 activation	+++	---	---	+++	+++	+++	+++
Casp-7 activation	+++	---	---	+++	---	---	+++
L. pneumophila replication	---	+++	+++	---	+++	+++	---
